# Methodology of Isfahan Tobacco Use Prevention Program: First Phase

**DOI:** 10.1155/2013/182170

**Published:** 2013-12-07

**Authors:** Hamidreza Roohafza, Kamal Heidari, Razieh Omidi, Tahereh Alinia, Fereshteh Rajabi, Saeid Bagheri, Rasoul Khormian Isfahani, Masoumeh Sadeghi

**Affiliations:** ^1^Tobacco Control Unit, Isfahan Cardiovascular Research Center, Isfahan Cardiovascular Research Institute, Isfahan University of Medical Sciences, P.O. Box 81465-1148, Isfahan, Iran; ^2^Isfahan Province Health Center, Isfahan University of Medical Sciences, Isfahan, Iran; ^3^Department of Epidemiology, Urmia University of Medical Sciences, West Azerbaijan, Iran; ^4^Health Unit, Isfahan Education Office, Isfahan, Iran; ^5^Expert Cultural and Prevention of Isfahan Drug Control Headquarters, Isfahan, Iran; ^6^Cardiac Rehabilitation Research Center, Isfahan Cardiovascular Research Institute, Isfahan University of Medical Sciences, Isfahan, Iran

## Abstract

*Background*. Tobacco use continues to be the leading global cause of preventable death. The majority of smokers begin using tobacco products at teen ages. The aims of this study were providing a methodology of Isfahan Tobacco Use Prevention Program and investigating the prevalence of tobacco use and its related factors. *Method*. It was a cross-sectional study among guidance and high school students in Isfahan province. Initiation, social, psychological (depression and self-efficacy), family, and attitudinal and belief factors and school policy toward smoking (cigarettes and water-pipe) were investigated. Saliva qutinin was given from 5% of participants for determination of accuracy of responses. A self-administered anonymous questionnaire was used for gathering all data. *Results*. Of all 5500 questionnaires distributed, about 5408 completed questionnaires were returned (with response rate of 98.3%). Of all participants, 2702 (50.0%) were girls and 2706 (50.0%) were boys. Respectively, 4811 (89.0%) and 597 (11.0%) were from urban and rural. Of all participants, 2445 (45.2%) were guidance school and 2962 (54.8%) were high school students. *Conclusion*. This study will provide a unique opportunity to study prevalence of smoking cigarettes and water-pipe (ghelyan) among guidance and high school students in Isfahan province and determine the role of initiation, social, psychological, family, and attitudinal and belief factors and school policy toward smoking.

## 1. Introduction

Tobacco use continues to be the leading global cause of preventable death and this disparity is expected to widen further over the next several decades. If current trends continue, by 2030 tobacco will kill more than 8 million people and cause hundreds of billions of dollars of economic damage worldwide each year, with 80% of these premature deaths among people living in low- and middle-income countries [1, 2].

Data on global tobacco use behavior shows that in many developed countries, the vast majority of smokers begin using tobacco products before the age of 18 years [3, 4]. There is some evidence in hand that, in Iran, more than one-third of ever smokers have tried their first cigarette before age 10. Recent trends indicate rising smoking prevalence rates among children and adolescents and earlier age of initiation. If these patterns continue, tobacco use will be the cause of 250 million children and adolescents deaths who are alive today, many of them in developing countries [4]. In addition to rising cigarette smoking, in Eastern Mediterranean Region (EMRO) another problem exists which is different from other parts of the world and it is the use of water-pipe, which is named ghelyan in Persian language and has become so popular especially among youth [5].

Data collected from different studies in Iran shows that about 12% of adults aged more than 15 years are smokers (25% males versus 1.4% females). According to Iran Global Youth Tobacco Survey (GYTS) report on 2007, 17.5% of youth in 13–15 years old age group have ever smoked cigarettes even 1 or 2 puffs. In this study 3% of 13–15 years old youth were current cigarette smokers. 26.1% of current smokers in this age group were using other types of tobacco products which mainly were water-pipe (ghelyan) that is raised compared to GYTS 2003. One of the important issues is that the percent of ever smokers tried their first cigarettes before age 10 is significantly raised from 2003 to 2007 (17.6% versus 36.1%) predominantly among boys (15.1% versus 40.7%) [4].

The behavior of tobacco experimentation is influenced by individual factors (perceptions, self-image, and peers) and various external factors as well, including physiological, psychological [6], social [7], and cultural factors and environmental factors such as advertising and economics [8]. Use of tobacco among friends, the experience of seeing others smoking at home or other places, receiving pocket money, receiving free tobacco from vendors, a poor school tobacco control environment, a high acceptance level of tobacco use, and exposure to advertisements and promotions of tobacco products were associated with a higher likelihood of adolescent tobacco use [9–13].

So that smoking is not only harmful to adolescents' physiological health, but it also damages their psychological health. Therefore, tobacco use and adolescent smoking have become a public health problem that urgently needs to be solved by reducing the overall smoking rate in the world.

Trying to control the preventable epidemic of tobacco using should be the first priority of the health administrators in all countries, especially developing countries. Implementing interventions to reduce tobacco use should be conducted with preventative implementations so treatments and preventive measures can be more effective in high-risk groups. To date, a few preventive measures have been conducted on tobacco control; so, first phase of Isfahan Tobacco Use Preventive Project (ITUPP) aimed to diagnose the prevalence of smoking in overall and to investigate cigarette and water-pipe (ghelyan) smoking motivators according to all related factors such as social, environmental, and cultural factors that influence initiation and use of tobacco among junior high school and high school students in Isfahan province.

## 2. Study Design

We conducted a cross-sectional study in Isfahan province among middle and high school students in September until October 2010. Isfahan is the second populated province in centre of Iran and has forty educational districts with more than 800,000 students. This study aimed to investigate the prevalence of smoking and determine factors related to smoking (cigarette or water-pipe (ghelyan)) among students.

Based on the study in 2003 that reported 14 percent of smoking among students [2] and considering 95 percent of confidence interval and 0.01 of error, sample size was calculated to be 5000. After considering attrition due to not answering or incomplete answering (defined as questionnaire that more than ten percent of items left blank), we estimated the sample size to be 5500.

Students were selected via a multistage random cluster sampling scheme to be a representative sample. Education districts were considered as clusters. Stratified sampling was taken based on the level (high/middle school), gender (boy/girl), and residency area (rural or urban area) within each cluster; then cluster sampling was conducted to randomly select schools among each cluster, at last random samples were taken from among selected schools using random numbers table. After enumeration, trained staff took informed consent from students after explanation of study design and goals and asked them to complete a questionnaire within 30 minutes during class time.

A workshop was set to train staff who was supposed to collect data and a protocol was designed to provide orders on how to complete the questionnaire and help students to fill out the questionnaire. They were also trained to take saliva samples from participants.

Saliva qutinin level is a good index for diagnosis of smoking. Its sensitivity and specificity is 96–97% and 99–100%, respectively [3]. Five percent of randomly selected students received a saliva vial. This action aimed to determine saliva qutinin based prevalence and compare it with self-reported prevalence for determination of accuracy of responses. Sample provided by rapid tongue and cheek movements at least half an hour after eating or drinking. It was replaced in the tube without touching. Staff transported saliva vials from the collection site in a cool bag and placed them in a freezer set at −20°C within four hours. The central laboratory of provincial health center analyzed samples using highly sensitive salivary qutinin quantitative ELIZA kite made in Austria. The cutoff point has been set to 48 ng/dL. The ethic committee of Isfahan University of Medical Science approved this study.

Four lecturers reviewed the questionnaire for its clarity, then 30 randomly selected students (15 females and 15 males) filled out the questionnaire. After completion of comments, Cronbach's alpha for internal consistency was evaluated and it was corrected for unrelated items.

## 3. Outcome Measurements

A self-administered anonymous questionnaire gathered data about some demographic and educational variables. These variables were age, gender (boy/girl), residency area (urban/rural), parents' educational level (0–5 y, 6–12 y, and >12 y), parents' occupation status (salaries-employed and self-employed worker, retired, unemployed, and housekeeper), students' number of educational years, average total score during last year, and number of absences in school (none, 1 to 3 days, 4 to 10 days, and more than 10 days).

Smoking status (for cigarette and water-pipe (ghelyan), separately) was classified into five subgroups. The strata were (1) never users have never tried, not even one puff, (2) those who tried at least one puff or more than one, (3) those who tried at least once a month but less than once a week, (4) those who tried at least once a week but less than once a day, and (5) at least once a day. First strata considered as never smoked, strata 2 as triers, and categories 3, 4, and 5 as smokers. Students answered a question on how many years they have smoked to compute age of smoking start and smoking duration.

### 3.1. Initiation Factors

Motivating factors of smoking initiation were explored using investigator invented questionnaire with nine-item dichotomous scale (Yes/No). The items consisted of the following: I smoke when (a) a bad event happens, (b) when I am angry, (c) when I am distressed and I believe it relaxes me, (d) when I am with friends to have fun, (e) when everyone smokes and it is a good entertainment, (f) after eating a meal, cause it is one of the best occasions for smoking, (g) as soon as I get up, cause it helps me to have a fine day, (h) because it decreases my appetite, and (i) when others smoke, as it encourages me to smoke.

### 3.2. Social Factors

*Peer smoking* was assessed by one item asking, “how many of your friends smoke (cigarette or water-pipe (ghelyan))?” (None of them, some of them, half of them, most of them, and all of them.) *Perceived social norms of smoking* were assessed by asking, “In your opinion, how many of the students in your age have been ever tried smoking, even for one puff?” (Less than 10%, 10–30%, 30–60%, 60–90%, and more than 90%.)

### 3.3. Family Factors

*Family smoking* was assessed by asking whether or not their father/mother or any brothers/sisters smoke cigarette or water-pipe (ghelyan). *Parental advice* was measured by asking how often your parents advise you not to smoke. Students were asked if they think that their parents are *able to prevent* them from smoking and if there is a *rule* in their house not to smoke inside. The answer of these question were assessed utilizing a 5-point Likert scale from 1 = definitely yes to 5 = definitely no. *Family conflict* was measured by a summary score of 3-item scale: (1) my parents nag me for any excuse, (2) my family does not understand me, and (3) I have a lot of argument with my family. The students could answer each item by having no idea, agreeing, disagreeing [14].

### 3.4. School Policy Toward Smoking

*School policy* toward smoking was covered by asking, does any of your teachers smoke on your school grounds (few, less than half, and nearly half, more than half, and nearly all), does your school have a clear set of rules about smoking (yes, no, and I do not know), how many students who smoke obey the rule of prohibiting smoking on school grounds (all of them, most of them, half of them, some of them, and none of them), to what extent do you think that school officials care about smoking behaviors of students (not at all, rarely, sometimes, often, and always)? In addition, the student's attitude toward school smoking policy was assessed. For this purpose, their idea (agreeing, having no idea, and disagreeing) was asked about these sentences: teachers are really trying to convince us not to smoke, teachers are really helping us how to refuse peer pressure to smoke, teachers and parents are really trying to make us a healthy place in school, and other students in my school put a lot of effort into stopping students from smoking at school.

### 3.5. Psychological Factors

*Refusal skills self-efficacy* was measured by asking, “Could you say no to a friend who offers you to smoke a cigarette or water-pipe (ghelyan)?” (Yes/No.) *Risk taking* was measured by a summary score of 3-item scale, (1) getting into trouble to have fun, worth, (2) I like risking and, (3) I enjoy doing things that people believe should not be done. Each item was assessed on a 5-point Likert scale where 1 = very well and 5 = not at all [14]. *Smoking intentions* were asked by asking, “Do you think you might try smoking cigarette or water-pipe (ghelyan) (or continue to smoke) in the future?” (Definitely yes, probably yes, probably no, and definitely no.) *Depression* as one of the underlying causes of smoking was evaluated in all of participants. For this purpose, they were asked by SCL-90 depression subscale to determine the item(s) that hurt them during the past four weeks. Loss of energy/sedentary, thoughts about death/suicidal ideation, easily fall to cry, feeling trapped, blaming yourself, loneliness, feeling of sadness, being excessive worried about everything, lack of interest for everything, sense of hopelessness about the future, feeling that anything is so difficult to do, and feeling worthlessness were the mentioned items. All responses are Yes/No and higher score indicates higher depression [15]. *Perceived self-efficacy* was assessed by using The General Self-Efficacy Scale consisting of 10 items. (1) I can always manage to solve difficult problems if I try hard enough. (2) If someone opposes me, I can find the means and ways to get what I want. (3) It is easy for me to stick to my aims and accomplish my goals. (4) I am confident that I could deal efficiently with unexpected events. (5) Thanks to my resourcefulness, I know how to handle unforeseen situations. (6) I can solve most problems if I invest the necessary effort. (7) I can remain calm when facing difficulties because I can rely on my coping abilities. (8) When I am confronted with a problem, I can usually find several solutions. (9) If I am in trouble, I can usually think of a solution. (10) I can usually handle whatever comes in my way. Responses on the 10-item scale range from 0 = not at all true to 3 = exactly true. Range of scores is 0–30 categorizing to three levels <15, 15–25, and >25 that, respectively, indicate low self-efficacy, moderate self-efficacy, and high self-efficacy [16].

### 3.6. Attitudinal and Belief Factors

*Attitudinal and belief factors* toward smoking were assessed by asking students attitude about nine items, using a 3-point response scale (agreeing, I have no idea, and disagreeing). (1) Sometimes, you feel you need to smoke a cigarette or even a puff of water-pipe (ghelyan). (2) Smoking is too expensive. (3) Children are more likely to smoke if their parents smoke. (4) Students should be allowed to smoke cigarette. (5) Sometimes, you like to show up as a smoker. (6) Smoking is something you do when other people want you to do it. (7) Smoking makes you feel grown up. (8) Smoking is hazardous to nonsmoker people's health. (9) Students should be allowed to smoke water-pipe (ghelyan).

## 4. Analysis Plan

The quantitative and qualitative variables will be expressed as Mean ± SD and Frequency (Percent). Prevalence of cigarettes smoking and water-pipe (ghelyan) will be estimated for total population as well as different levels of sociodemographic variables of study participants. The association between smoking and initiation, social, family attitude, and belief factors in line with the main objectives of our study will be investigated.

A normal distribution will be checked using the Kolmogorov-Smirnov test and p-p plot. Differences based on continuous data with normal distribution will be analyzed using an independent samples *t*-test in univariate and multivariate analysis variance (MANOVA) settings, respectively. In this regards, analysis of covariance (ANCOVA) and multivariate analysis of covariance (MANCOVA) will be used for adjusting the potential continuous covariates, as appropriate. In both approaches Bonferroni post-hoc test will be used for pairwise comparisons. Mann-Whitney *U*-test, as a counterpart of independent samples *t*-test, for nonnormally distributed data will be applied. Discriminant analysis (DA) will be conducted for identifying the most effective continuous discriminative factors on smoking behaviors.

Association between categorical data (irrespective of dependent and independent role of variables) will be analyzed using Chi-square test and log-linear models. The association between categorical (binary, nominal, and ordinal) dependent variables and independent factors will be analyzed using binary, multinomial, and ordinal logistic regression models. Survey data will be coded and analyzed using the Statistical Package for the Social Sciences (SPSS 15). A *P* value <0.05 will be considered as statistically significant level.

## 5. Results

Before the final version of the questionnaire was adopted for use in present study, a pilot study was conducted where the questionnaires were administered to a group of 30 students' school to assess their reliability. The results have been shown in Table 1.

After evaluating internal consistency of all questionnaires, 5500 questionnaires were distributed and 5408 completed questionnaires were returned (with response rate of 98.33%). Of all participants 2702 (50.0%) were girls and 2706 (50.0%) were boys. Respectively, 4811 (89.0%) and 597 (11.0%) were from urban and rural. Of all participants 2445 (45.2%) were guidance school and 2962 (54.8%) were high school students. Five hundred and seventy three (11.4%), 661 (13.2%), and 981 (19.6%) were first, second, and third grade of guidance school and 1050 (21.0%), 895 (17.9%), 657 (13.1%), and 189 (3.7%) were first, second, third, and fourth grade of high school students. Tables 2 and 3 show the distribution of population based on sex and residency.

## 6. Discussion

The Isfahan Tobacco Use Prevention Program has 3 phases. The aim of this phase 1 study is to provide a methodology to diagnose the targeted community in order to investigate the prevalence of smoking and smoking motivators according to all related factors among junior high school and high school students in Isfahan province.

The response rate in our study was satisfactory. Cooperating teachers in conducting the study, providing enough information to subject about how to fulfill the questionnaires, and using incentives were the main reason for the acceptable rate of participation in our study.

The main advantage of a multicluster random sampling school-based epidemiological approach is the opportunity to obtain valuable information about target population that helps us to design interventions for reducing school-based problems.

This study will provide a unique opportunity to study prevalence of smoking cigarettes and water-pipe (ghelyan) among guidance and high school students and determine the role of initiation, social, psychological, family, and attitudinal and belief factors and school policy toward smoking.

We hope such valuable data that are provided through this study can help us in developing public health policy and regional strategies in preventing tobacco use among adolescents.

Given that initiation, social, psychological, family, and attitudinal and belief factors and school policy toward smoking can be an effect on initiation and continuing tobacco use; consequently, investigating effective factors of tobacco use in adolescents is very important. In addition prevention and interventional programs aimed at reducing teen tobacco experimentation can be conducted at home and school with support by parents, peers, and teachers. Our findings should prove useful for future development of intervention strategies among adolescents in Iran.

## Figures and Tables

**Table 1:** Internal consistency of all questionnaires.

Questionnaire	Cronbach's alpha	N of items
Smoking initiation	0.77	9
Family conflict	0.73	3
Risk taking	0.71	3
Depression (SCL-90 subscale)	0.88	12
General self efficacy	0.86	10
Attitude toward smoking	0.79	9

**Table 2:** Distribution of the population based on sex.

Sex		
	Boy	Girl
Urban	2401 (49.9)	2410 (50.1)
Rural	305 (51.1)	292 (48.9)
Guidance school	1217 (49.8)	1228 (50.2)
High school	1488 (50.2)	1474 (49.8)
Grade 6	222 (38.7)	351 (61.3)
Grade 7	314 (47.5)	347 (52.5)
Grade 8	533 (54.3)	448 (45.7)
Grade 9	550 (52.4)	500 (47.6)
Grade 10	480 (53.6)	415 (46.4)
Grade 11	288 (43.8)	369 (56.2)
Grade 12	74 (39.1)	115 (60.9)

**Table 3:** Distribution of the population based on residency.

Residency		
	Rural	Urban
Girl	292 (10.8)	2410 (89.2)
Boy	305 (11.3)	2401 (88.7)
Guidance school	380 (15.5)	2065 (84.5)
High school	217 (7.3)	2745 (92.7)
Grade 6	75 (13.1)	498 (86.9)
Grade 7	107 (16.2)	554 (83.8)
Grade 8	140 (14.3)	841 (85.7)
Grade 9	101 (9.6)	949 (90.4)
Grade 10	64 (7.2)	831 (92.8)
Grade 11	36 (5.5)	621 (94.5)
Grade 12	18 (10.2)	171 (89.8)
